# Mesenchymal stem cell-specific Sirt1 overexpression prevents sarcopenia induced by 1,25-dihydroxyvitamin D deficiency

**DOI:** 10.18632/aging.206232

**Published:** 2025-03-31

**Authors:** Haiyun Chen, Biqi Ren, Jing Wang, Xingchen Liu, Xiangjiao Yi, David Goltzman, Dengshun Miao

**Affiliations:** 1 Department of Plastic Surgery, Affiliated Friendship Plastic Surgery Hospital of Nanjing Medical University, Nanjing Medical University, Nanjing, China; 2 Department of Topographic Anatomy, Basic Medical College, Jiamusi University, Jiamusi, Heilongjiang, People's Republic of China; 3 Department of Human Anatomy, Histology and Embryology, The Research Center for Bone and Stem Cells, Nanjing Medical University, Nanjing, Jiangsu, People's Republic of China; 4 Institute of Orthopaedics and Traumatology, The First Affiliated Hospital of Zhejiang Chinese Medical University (Zhejiang Provincial Hospital of Traditional Chinese Medicine), Hangzhou, Zhejiang, People's Republic of China; 5 Calcium Research Laboratory, McGill University Health Centre and Department of Medicine, McGill University, Montreal, Quebec H4A 3J1, Canada

**Keywords:** active vitamin D, muscle regeneration, Myod1, sarcopenia, Sirt1

## Abstract

Sarcopenia, characterized by an age-related decline in skeletal muscle mass and function, is closely linked to vitamin D deficiency. This study examines the role of Sirtuin 1 (Sirt1) and its regulation by vitamin D in preventing sarcopenia. Utilizing wild-type, 1α-hydroxylase knockout (1α(OH)ase^−/−^), and Sirt1 transgenic (Sirt1^Tg^) 1α(OH)ase^−/−^ mice, we investigated muscle Sirt1 levels, muscle mass, fiber type, and senescence markers. Our results demonstrated that 1,25-Dihydroxyvitamin D (1,25(OH)2D3) upregulated Sirt1 and myogenic factor MyoD1 expression in C2C12 myoblasts via VDR-mediated transcription. Sirt1 overexpression in mesenchymal stem cells (MSCs) significantly mitigated muscle mass reduction, improved fiber cross-sectional area, and increased type II fiber numbers in 1α(OH)ase^−/−^ mice. Mechanistically, 1,25(OH)2D3 promoted muscle cell health by enhancing Sirt1 expression, which in turn reduced muscle cell senescence and the senescence-associated secretory phenotype (SASP) through decreased levels of acetylated nuclear p53 and p65, maintaining their cytoplasmic localization. Additionally, Sirt1 overexpression accelerated muscle regeneration post-injury by increasing embryonic myosin heavy chain expression and cell proliferation. These findings underscore the therapeutic potential of targeting vitamin D and Sirt1 pathways to prevent sarcopenia, suggesting that supplementation with active vitamin D and consequent Sirt1 activation could be effective strategies for managing age-related muscle wasting.

## INTRODUCTION

Sarcopenia is a progressive skeletal muscle disorder characterized by reduced mass, strength, and function, recognized as a disease by the World Health Organization [1]. This condition poses significant health risks in the elderly, including falls, fractures, frailty, and dependence, leading to decreased quality of life and adverse health outcomes [2]. The prevalence of sarcopenia among older adults is substantial, and as the global population ages, it becomes crucial to investigate its origins and develop effective management strategies [3].

The pathogenesis of sarcopenia involves various factors, including age, endocrine dysfunction, malnutrition, inflammation, and vitamin D deficiency [4]. Vitamin D deficiency is commonly associated with aging, endocrine disorders, malnutrition, and inflammation [5–7]. Clinical and epidemiological evidence supports the impact of vitamin D on muscle function, with decreased vitamin D levels being associated with reduced muscle parameters and increased fall risk [8, 9]. Vitamin D supplementation has been shown to improve muscle function and balance [10]. Therefore, understanding the relationship between vitamin D and sarcopenia is of great importance.

Recent clinical studies demonstrated that low serum vitamin D levels were significantly associated with a higher prevalence of sarcopenia in a large cohort of elderly individuals and have provided valuable insights into the association between vitamin D and sarcopenia [11]. Another study reported that vitamin D supplementation improved muscle strength and physical performance in sarcopenic older adults [12]. These findings highlight the clinical relevance of vitamin D in the prevention and management of sarcopenia.

The vitamin D receptor (VDR) is expressed in muscle fibers, and the active form of vitamin D, 1,25-dihydroxyvitamin D (1,25(OH)2D), regulates myoblast proliferation and differentiation through VDR signaling [13]. However, the precise mechanisms linking vitamin D deficiency to sarcopenia and its impact on muscle regeneration remain unclear.

In this study, we aimed to investigate the role of vitamin D and its interaction with Sirtuin 1 (Sirt1) in the development of sarcopenia. Sirt1 is a longevity gene known to aid in muscle repair and metabolic regulation, playing a role in inhibiting muscle atrophy and promoting muscle metabolism [14, 15]. Recent studies have shown that Sirt1 overexpression in mesenchymal stem cells (MSCs) can prevent osteoporosis in mice deficient in 1α-hydroxylase, the enzyme responsible for converting inactive vitamin D to its active form [16]. Therefore, we hypothesized that 1,25(OH)2D upregulates Sirt1 in myoblasts via VDR signaling and that Sirt1 overexpression in MSCs may prevent sarcopenia induced by 1,25(OH)2D deficiency.

To test this hypothesis, we conducted experiments using wild-type, 1α-hydroxylase knockout (1α(OH)ase^−/−^), and Sirt1 transgenic (Sirt1^Tg^) 1α(OH)ase^−/−^ mice. We examined Sirt1 expression levels in muscle tissues and investigated the regulation of Sirt1 by 1,25(OH)2D in myoblasts through VDR-mediated gene transcription. Additionally, we evaluated the effects of Sirt1 overexpression in MSCs on muscle mass, fiber type, senescence markers, and muscle regeneration in the context of 1,25(OH)2D deficiency-induced sarcopenia.

This study not only contributes to our understanding of the mechanisms underlying the relationship between vitamin D and sarcopenia but also reveals the potential of vitamin D and Sirt1 activation as therapeutic targets for the prevention and management of sarcopenia. By incorporating the latest clinical data on vitamin D and sarcopenia [17], our research aims to provide valuable insights and evidence-based strategies to attenuate age-related muscle wasting.

## RESULTS

### 1,25(OH)_2_D_3_ upregulates Sirt1 expression in C2C12 cells via VDR-mediated gene transcription

**Figure 1:**
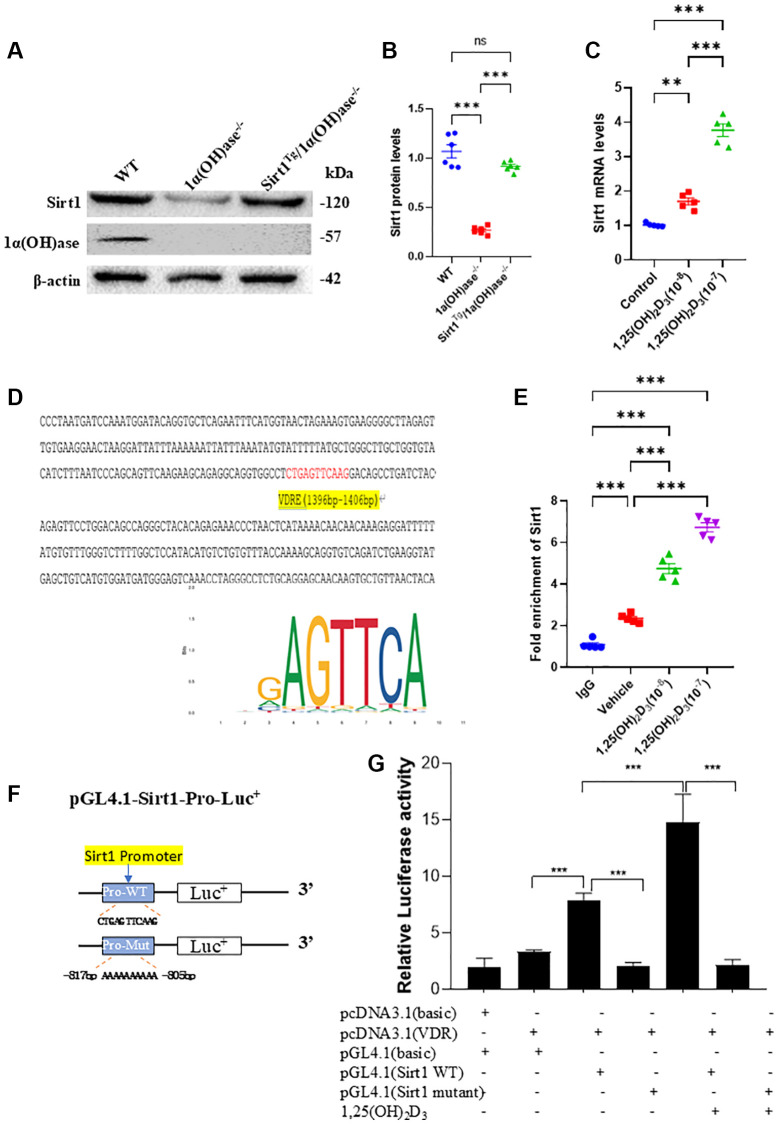
**1,25(OH)_2_D_3_ upregulates Sirt1 expression in C2C12 cells via VDR-mediated transcription.** (**A**) Western blot showing changes in Sirt1 and 1α(OH)ase protein expression levels in tibialis anterior muscle tissues of 2-month-old WT, 1α(OH)ase^−/−^ and Sirt1^Tg^1α(OH)ase^−/−^ mice and (**B**) statistical analysis results of Sirt1 protein expression levels. (**C**) 1,25(OH)_2_D_3_ treatment upregulated Sirt1 gene expression in C2C12 cells. (Each experiment was conducted in five replicates). (**D**) VDR binding sites were predicted in the Sirt1 promoter region (yellow area). (**E**) ChIP-qPCR results showing enrichment of Sirt1 in VDR immunoprecipitation. (**F**) Schematic diagram of luciferase reporter gene plasmids containing the Sirt1 promoter region. (**G**) Luciferase activity results. ^**^p < 0.01; ^***^p < 0.001.

Our previous studies showed that Sirt1 protein expression was significantly decreased in the skeletal system of 1α(OH)ase^−/−^ mice. In this study, we used Western blotting to detect changes in Sirt1 protein expression levels in tibialis anterior muscle of 2-month-old male wild-type, 1α(OH)ase^−/−^ and Sirt1^Tg^1α(OH)ase^−/−^ littermate mice. 1α(OH)ase protein expression was detected in tibialis anterior muscle of wild-type mice, but not in 1α(OH)ase^−/−^ and Sirt1^Tg^1α(OH)ase^−/−^ littermate mice. Sirt1 protein expression was significantly downregulated in skeletal muscle tissues of 1α(OH)ase^−/−^ mice, and upregulated in skeletal muscle tissues of Sirt1^Tg^1α(OH)ase^−/−^ mice (Figure 1A, 1B). After treating C2C12 cells with 10^−8^ or 10^−7^ M 1,25(OH)_2_D_3_, real-time RT-PCR detected a dose-dependent upregulation of Sirt1 mRNA expression (Figure 1C). Bioinformatics analysis predicted a VDR binding site in the Sirt1 gene promoter region (Figure 1D). To confirm that the predicted VDR binding site in the Sirt1 gene promoter region was sufficient to promote Sirt1 transcription, we used ChIP to detect whether VDR could bind to the Sirt1 promoter. PCR detection of the ChIP products showed that the Sirt1 promoter sequence was present in the total sample, and VDR antibody immunoprecipitation enriched the Sirt1 promoter region more than IgG immunoprecipitation (Figure 1E). To further demonstrate that 1,25(OH)_2_D_3_ regulates Sirt1 gene expression via VDR-mediated transcription, we constructed luciferase reporter plasmids containing the Sirt1 promoter region and a VDR overexpression plasmid. Transfection into C2C12 cells followed by 1,25(OH)_2_D_3_ treatment and luciferase reporter assay after 48 hours showed that compared to empty plasmid, luciferase activity significantly increased in C2C12 cells transfected with pGL4.1-Sirt1 (WT) plasmid, and increased even more significantly in 1,25(OH)_2_D_3_ treated C2C12 cells. However, luciferase activity did not increase in C2C12 cells transfected with pGL4.1-Sirt1 (mutant) plasmid, and 1,25(OH)_2_D_3_ treatment also failed to activate the mutant reporter (Figure 1F, 1G). These results demonstrate that 1,25(OH)_2_D_3_ upregulates Sirt1 gene expression in C2C12 cells via VDR-mediated transcription.

### Sirt1 overexpression in MSCs corrects skeletal muscle mass reduction, muscle fiber atrophy and type II fiber loss caused by 1,25(OH)_2_D deficiency

**Figure 2:**
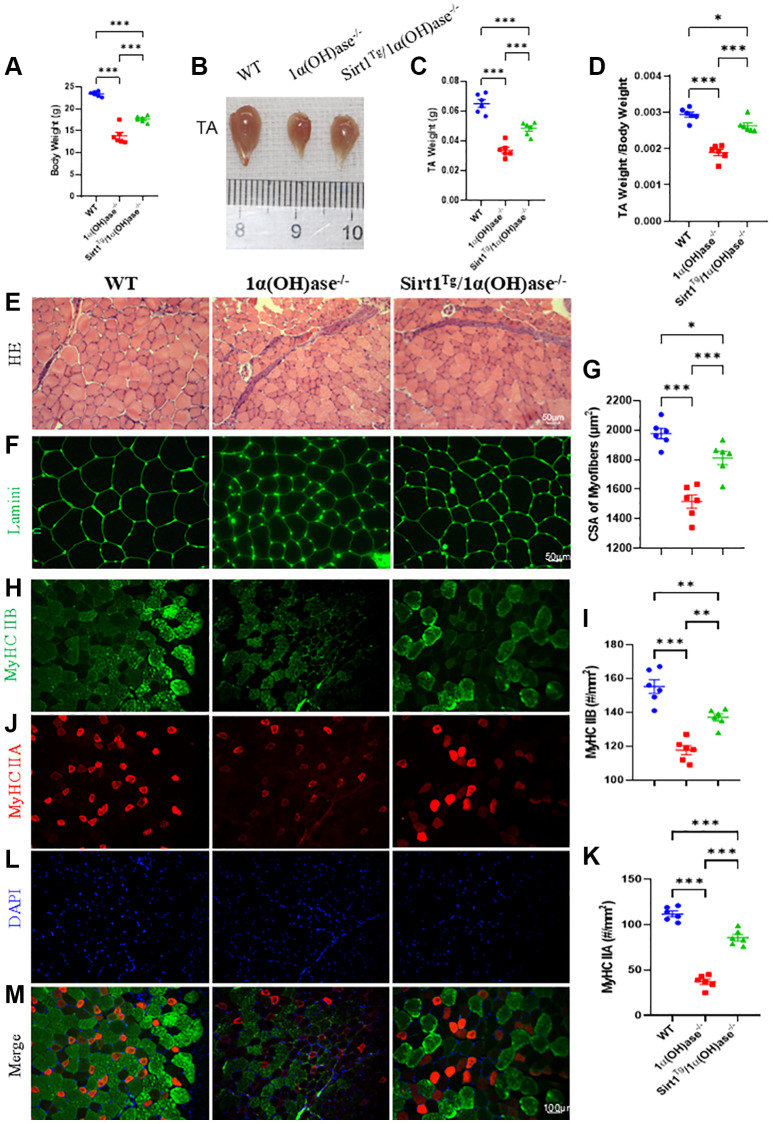
**Sirt1 overexpression in MSCs corrects skeletal muscle mass reduction, muscle fiber atrophy and type II fiber loss caused by 1,25(OH)_2_D deficiency.** (**A**) Body weight results, (**B**) gross images of tibialis anterior (TA) muscles and (**C**) muscle weight results. (**D**) Tibialis anterior muscle weight/body weight results from 2-month-old WT, 1α(OH)ase^−/−^ and Sirt1^Tg^1α(OH)ase^−/−^ male mice. (**E**) H&E staining micrographs. (**F**) Laminin immunofluorescence staining micrographs. (**G**) Tibialis anterior muscle cross-sectional area analysis results. (**H**) Tibialis anterior muscle MyHC II B immunofluorescence staining micrographs and (**I**) MyHC II B positive fiber number analysis. (**J**) Tibialis anterior muscle MyHC II A immunofluorescence staining micrographs and (**K**) MyHC II A positive fiber number analysis. (**L**) DAPI staining of tibialis anterior muscle sections. (**M**) Merged images of MyHC II B, MyHC II A and DAPI immunofluorescence staining in tibialis anterior muscle. 6 mice per group were used for experiments. ^*^p < 0.05; ^**^p < 0.01; ^***^p < 0.001.

To determine whether Sirt1 overexpression in MSCs could correct skeletal muscle mass reduction caused by 1,25(OH)_2_D deficiency, we measured body weight, tibialis anterior muscle weight, and tibialis anterior muscle weight relative to body weight in 2-month-old male wild-type, 1α(OH)ase^−/−^ and Sirt1^Tg^1α(OH)ase^−/−^ littermates. Statistical analysis showed that body weight, tibialis anterior muscle weight, and tibialis anterior muscle weight/body weight ratio were significantly lower in 1α(OH)ase^−/−^ mice compared to wild-type mice. In contrast, they were significantly higher in Sirt1^Tg^1α(OH)ase^−/−^ mice compared to 1α(OH)ase^−/−^ mice (Figure 2A–2D). These results demonstrate that 1,25(OH)_2_D deficiency can lead to skeletal muscle mass reduction, while Sirt1 overexpression in MSCs can significantly improve skeletal muscle mass reduction caused by 1,25(OH)_2_D deficiency.

To determine whether the improvement in skeletal muscle mass reduction caused by 1,25(OH)_2_D deficiency by Sirt1 overexpression in MSCs is related to changes in muscle fiber cross-sectional area and type II fiber number, we collected tibialis anterior muscle from 2-month-old male wild-type, 1α(OH)ase^−/−^ and Sirt1^Tg^1α(OH)ase^−/−^ littermates. Muscles were rapidly frozen in pre-cooled isopentane using liquid nitrogen, embedded in OCT, and cryosectioned. Sections were stained with H&E and laminin immunofluorescence and quantified using ImageJ. The results showed that muscle fiber cross-sectional area was significantly smaller in 1α(OH)ase^−/−^ mice compared to wild-type mice, while Sirt1 overexpression in MSCs significantly increased the reduced tibialis anterior muscle fiber cross-sectional area caused by 1,25(OH)_2_D deficiency (Figure 2E–2G). MyHC IIA and MyHC IIB immunofluorescence staining showed that compared to wild-type mice, MyHC IIA and MyHC IIB positive muscle fibers were significantly reduced in 1α(OH)ase^−/−^ mice. Compared to 1α(OH)ase^−/−^ mice, MyHC IIA and MyHC IIB positive muscle fiber numbers were markedly increased in Sirt1^Tg^1α(OH)ase^−/−^ mice (Figure 2H–2M). These results indicate that Sirt1 overexpression in MSCs can improve skeletal muscle mass reduction caused by 1,25(OH)_2_D deficiency by correcting muscle fiber atrophy and type II fiber loss.

### Sirt1 overexpression in MSCs corrects satellite cell reduction and skeletal muscle cell senescence caused by 1,25(OH)_2_D deficiency

**Figure 3:**
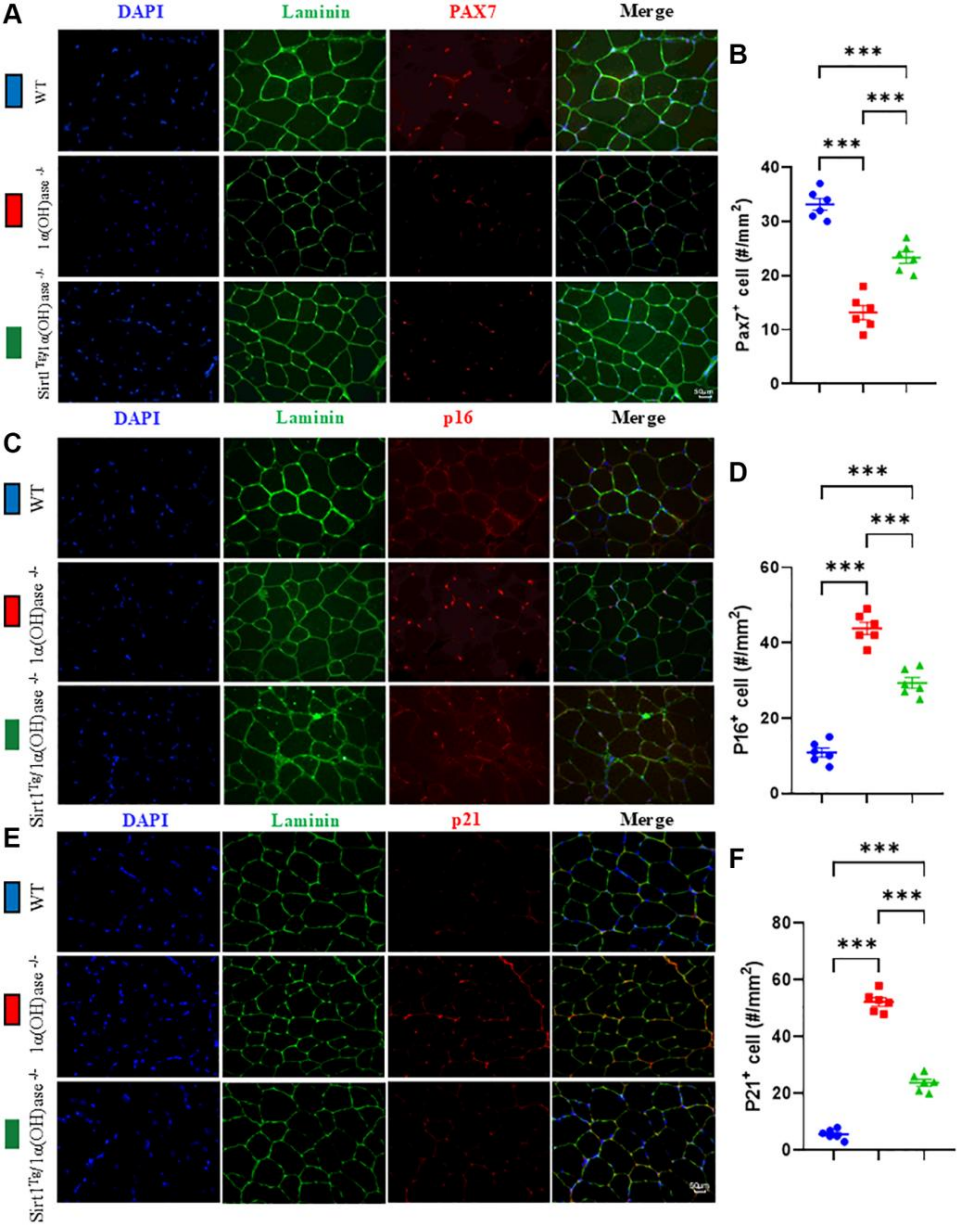
**Sirt1 overexpression in MSCs corrects satellite cell reduction and skeletal muscle cell senescence caused by 1,25(OH)_2_D deficiency.** (**A**) DAPI, Laminin and Pax7 immunofluorescence staining micrographs of tibialis anterior muscle from 2-month-old WT, 1α(OH)ase^−/−^ and Sirt1^Tg^1α(OH)ase^−/−^ male mice. (**B**) Statistical analysis of Pax7 positive cells in tibialis anterior muscle. (**C**) DAPI, Laminin and p16 immunofluorescence staining micrographs. (**D**) p16 positive cell analysis results. (**E**) DAPI, Laminin and p21 immunofluorescence staining micrographs from tibialis anterior muscle of 2-month-old mice of three genotypes. (**F**) p21 positive cell analysis results. 6 mice per group were used for experiments. ^*^p < 0.05, ^**^p < 0.01, ^***^p < 0.001.

To determine whether the correction of 1,25(OH)_2_D deficiency-induced sarcopenia by Sirt1 overexpression in MSCs is related to inhibition of the reduction in satellite cell numbers in skeletal muscle caused by 1,25(OH)_2_D deficiency, we collected tibialis anterior muscle from 2-month-old male wild-type, 1α(OH)ase^−/−^ and Sirt1^Tg^1α(OH)ase^−/−^ mice, and performed Pax7 immunofluorescence staining on cryosections to compare changes in skeletal muscle satellite cell numbers between genotypes. The results showed that the percentage of Pax7 positive cells was significantly decreased in tibialis anterior muscle of 1α(OH)ase^−/−^ mice compared to wild-type mice. The percentage of Pax7 positive cells was significantly increased in tibialis anterior muscle of Sirt1^Tg^1α(OH)ase^−/−^ mice compared to 1α(OH)ase^−/−^ mice (Figure 3A, 3B). These results indicate that Sirt1 overexpression in MSCs may improve 1,25(OH)_2_D deficiency-induced sarcopenia by increasing satellite cell numbers.

To determine whether the correction of 1,25(OH)_2_D deficiency-induced sarcopenia by Sirt1 overexpression in MSCs is related to inhibition of skeletal muscle cell senescence caused by 1,25(OH)_2_D deficiency, we collected tibialis anterior muscle from 2-month-old wild-type, 1α(OH)ase^−/−^ and Sirt1^Tg^1α(OH)ase^−/−^ mice and performed p16 and p21 immunofluorescence staining on cryosections to compare changes in skeletal muscle cell senescence between genotypes. The results showed that the percentage of p16 and p21 positive cells was significantly increased in 1α(OH)ase^−/−^ mice compared to wild-type mice. In contrast, these markers were significantly decreased in Sirt1^Tg^1α(OH)ase^−/−^ mice compared to 1α(OH)ase^−/−^ mice (Figure 3C–3F). These results indicate that Sirt1 overexpression in MSCs may correct 1,25(OH)_2_D deficiency-induced sarcopenia by inhibiting skeletal muscle cell senescence.

### Sirt1 overexpression in MSCs corrects senescence-associated secretory phenotype (SASP) in skeletal muscle caused by 1,25(OH)_2_D deficiency

**Figure 4:**
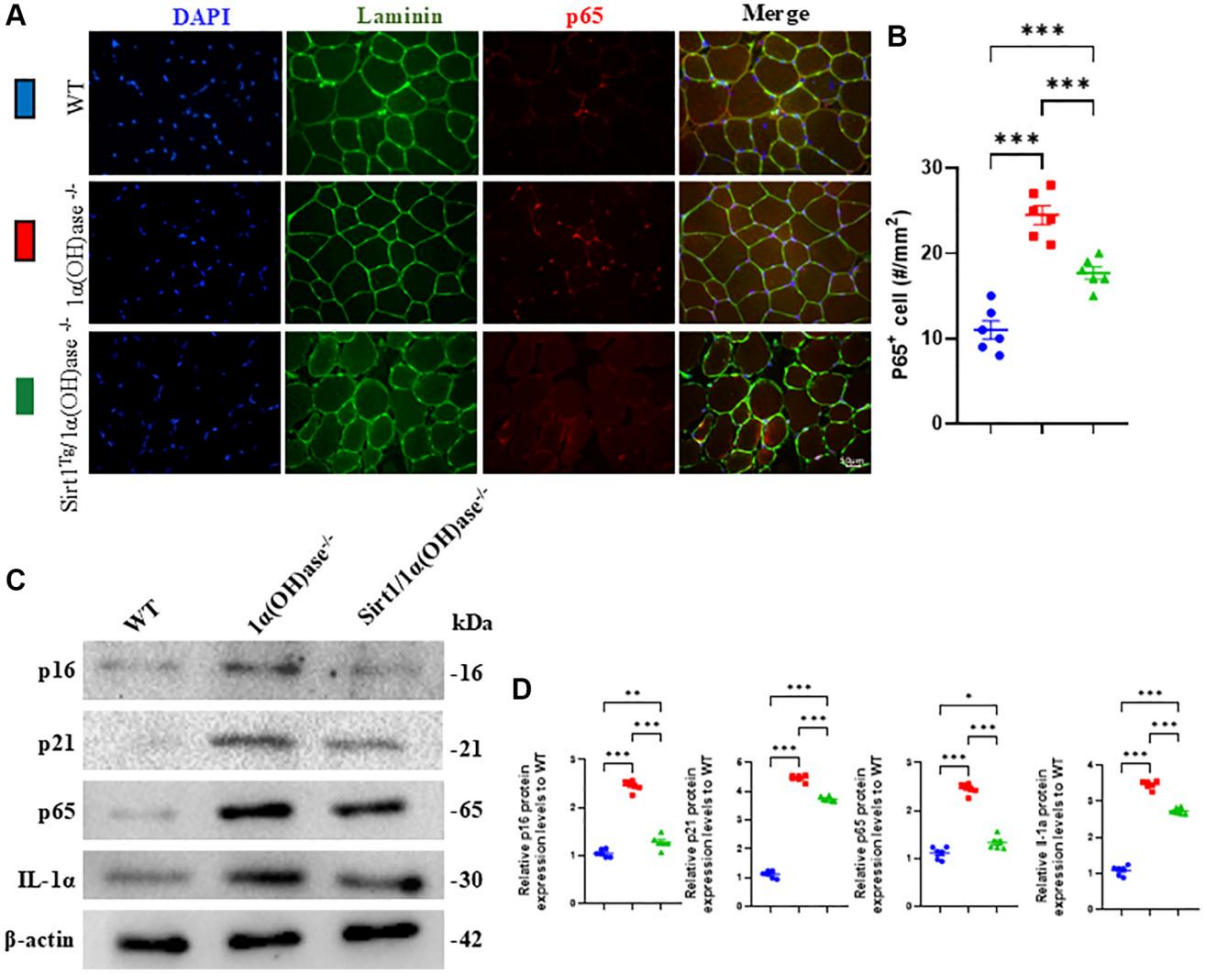
**Sirt1 overexpression in MSCs corrects senescence-associated secretory phenotype (SASP) in skeletal muscle caused by 1,25(OH)_2_D deficiency.** (**A**) DAPI, Laminin and p65 immunofluorescence staining micrographs of tibialis anterior muscle from 2-month-old male mice of three genotypes and (**B**) p65 positive cell analysis results. (**C**) Western blot showing changes in p16, p21, p65 and IL-1α protein levels in tibialis anterior muscle and (**D**) quantitative analysis of protein levels. 6 mice per group were used for experiments. ^*^p < 0.05; ^**^p < 0.01; ^***^p < 0.001.

To determine whether the correction of 1,25(OH)_2_D deficiency-induced sarcopenia by Sirt1 overexpression in MSCs is related to inhibition of increased skeletal muscle SASP caused by 1,25(OH)_2_D deficiency, we collected tibialis anterior muscle from 2-month-old mice of the three genotypes and used p65 immunofluorescence staining and Western blots to compare changes in skeletal muscle cell senescence and SASP-related markers between genotypes. The results showed that the percentage of p65 positive cells and the protein expression levels of p16, p21, p65 and IL-1α were significantly increased in 1α(OH)ase^−/−^ mice compared to wild-type mice. In contrast, these markers were significantly decreased in Sirt1^Tg^1α(OH)ase^−/−^ mice compared to 1α(OH)ase^−/−^ mice (Figure 4A–4D). These results indicate that Sirt1 overexpression in MSCs may correct 1,25(OH)_2_D deficiency-induced sarcopenia by inhibiting skeletal muscle SASP.

### 1,25(OH)_2_D_3_ or resveratrol inhibits C2C12 cell senescence and SASP by activating Sirt1 to decrease acetylated p53 and p65 levels and activities

**Figure 5:**
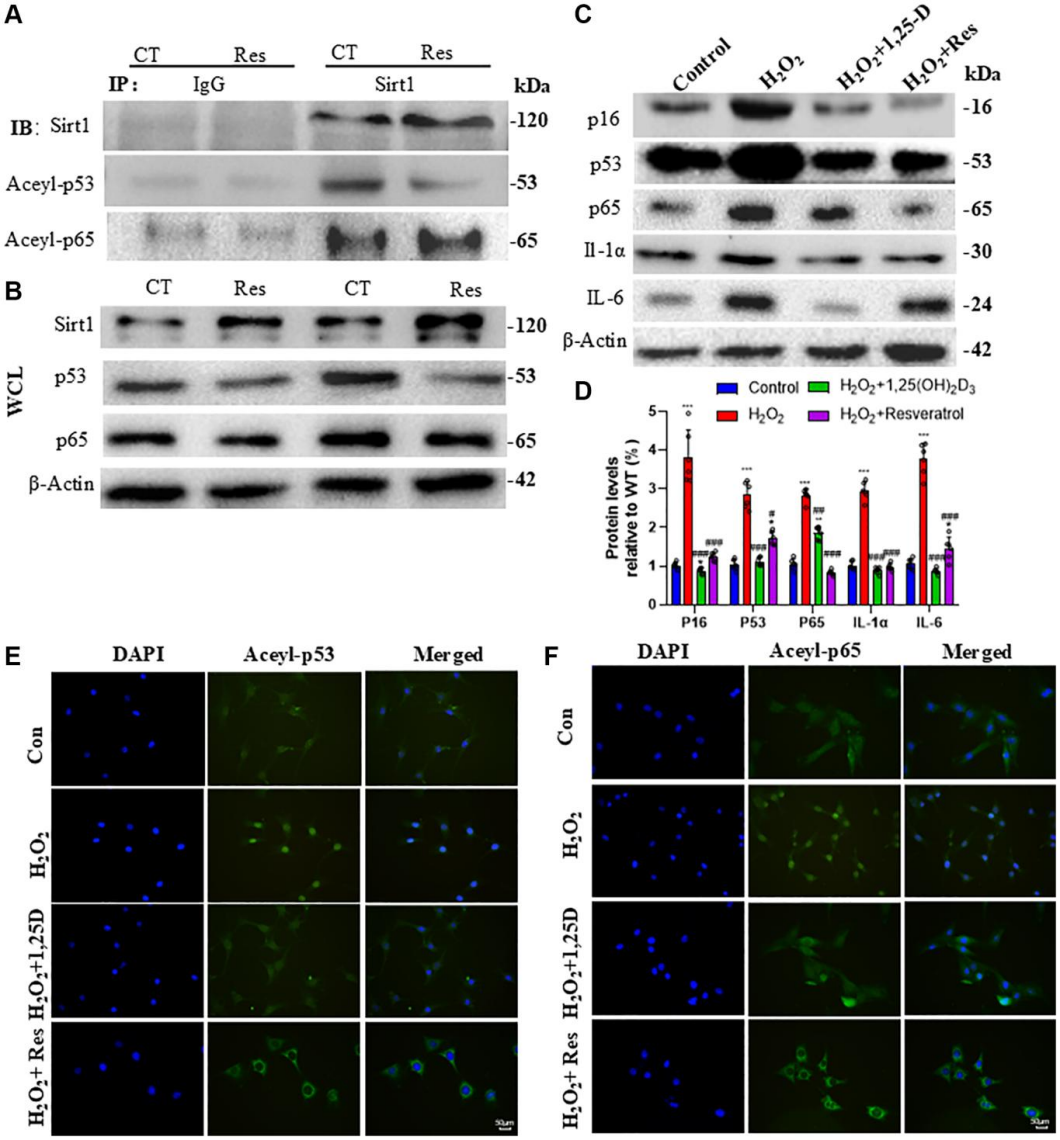
**1,25(OH)_2_D_3_ or resveratrol inhibits C2C12 cell senescence and SASP by activating Sirt1 to decrease acetylated p53 and p65 levels and activities.** (**A**) C2C12 cells were treated with 10 µM Resveratrol (Res) and Sirt1 protein immunoprecipitation followed by Western blot analysis of Sirt1, Acel-p53 and Aceyl-p65 expression. (**B**) Western blot analysis of Sirt1, p53 and p65 protein levels in whole cell lysates (WCL). (**C**) C2C12 cells were treated with 100 µM H_2_O_2_ alone or together with 10^−8^M 1,25(OH)_2_D_3_ or 10 µM Resveratrol for 24 hours, proteins were extracted and p16, p53, p65, IL-1α and IL-6 levels were determined by Western blot. (**D**) Quantitative analysis of protein levels. Experiments were performed in triplicate. ^*^p < 0.05; ^***^p < 0.001 compared to control; ^#^*p* < 0.05; ^##^*p* < 0.01; ^###^*p* < 0.001 compared to H_2_O_2_ alone. (**E**) Immunofluorescence staining showing subcellular localization of Acel-p53. (**F**) Immunofluorescence staining showing subcellular localization of Aceyl-p65.

To determine if resveratrol decreases acetylated p53 and p65 levels by activating Sirt1, we treated C2C12 cells with 10 µM resveratrol for 24 hours, immunoprecipitated proteins with anti-Sirt1 versus IgG control, and performed Western blots to detect changes in Sirt1, acetylated p53, acetylated p65, along with Sirt1, p53 and p65 in whole cell lysates (WCL). The results showed Sirt1 could physically bind to p53 and p65 in C2C12 cells, and resveratrol treatment enhanced Sirt1 binding to them and decreased acetylated p53 and p65 levels (Figure 5A). WCL Western blots showed resveratrol markedly increased Sirt1 expression and decreased p53 and p65 expression in C2C12 cells (Figure 5B).

To determine if 1,25(OH)_2_D_3_ or resveratrol can inhibit oxidative stress-induced senescence and SASP in skeletal muscle cells, we pretreated C2C12 cells with 100 µM H_2_O_2_ for 6 hrs followed by 10^−8^M 1,25(OH)_2_D_3_ or 10 µM resveratrol for 24 hrs, and performed Western blots. The results showed that H_2_O_2_ significantly increased p16, p53, p65, IL-1α and IL-6 protein levels, which were decreased by 1,25(OH)_2_D_3_ or resveratrol (Figure 5C, 5D). To further determine if the inhibition of H_2_O_2_-induced senescence and SASP by 1,25(OH)_2_D_3_ and resveratrol were associated with altered subcellular localization of acetylated p53 and p65, we performed immunofluorescence staining. The results showed acetylated p53 and p65 were mainly nuclear in H_2_O_2_-treated C2C12 cells, but were largely cytoplasmic after 1,25(OH)_2_D_3_ or resveratrol treatment (Figure 5E, 5F). Together, these results support the hypothesis that 1,25(OH)_2_D_3_ or resveratrol can inhibit oxidative stress-induced senescence and SASP in C2C12 cells by activating Sirt1 to decrease acetylated nuclear p53 and p65 levels and activities.

### Sirt1 overexpression in MSCs accelerates skeletal muscle injury repair by promoting regeneration

**Figure 6:**
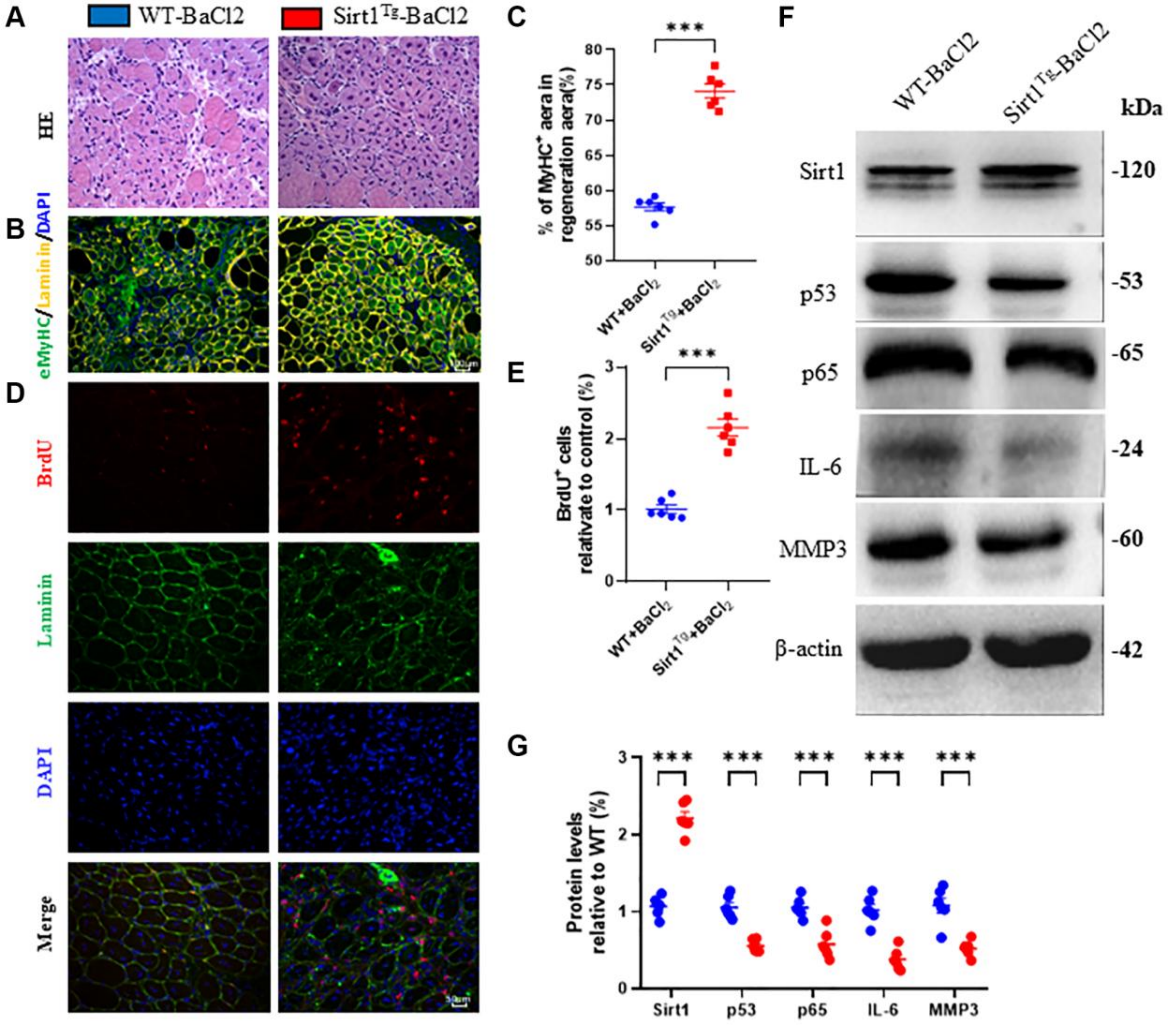
**Sirt1 overexpression in MSCs accelerates skeletal muscle injury repair by promoting regeneration.** (**A**) H&E staining micrographs of tibialis anterior muscle frozen sections from 2-month-old WT and Sirt1^Tg^ mice after barium chloride (BaCl2) injury. (**B**) eMyHC/Laminin/DAPI immunofluorescence staining micrographs and (**C**) relative eMyHC^+^ newborn fiber area analysis. (**D**) BrdU/Laminin/DAPI immunofluorescence staining micrographs and (**E**) BrdU positive fiber number analysis. (**F**) Western blot analysis of Sirt1, p53, p65, IL-6 and Mmp3 protein levels in muscle tissues after BaCl2 injury and (**G**) quantitative protein analysis. 6 mice per group were used for experiments. ^*^p < 0.05; ^**^p < 0.01; ^***^p < 0.001 compared to WT + BaCl2.

To determine if Sirt1 overexpression in MSCs can promote skeletal muscle regeneration and accelerate injury repair, we injured the right tibialis anterior muscle of 2-month-old wild-type and Sirt1^Tg^ mice with BaCl_2_. After 5 days, injured muscles were collected for cryosection and immunofluorescence staining of BrdU injected intraperitoneally 48 hrs before tissue collection. H&E staining, eMyHC, and BrdU immunofluorescence staining were used to analyze muscle injury repair. Western blots were performed on tissue lysates. Compared to WT+BaCl_2_, the number of newborn muscle fibers, eMyHC positive fiber area, and percentage of BrdU positive cells were significantly increased at 5 days after injury in Sirt1^Tg^+BaCl_2_ mice (Figure 6A–6E). Western blots of injured muscle lysates showed Sirt1 protein expression was significantly increased, while p53, p65, IL-6 and Mmp3 levels were decreased in Sirt1^Tg^+BaCl_2_ compared to WT+BaCl_2_ mice (Figure 6F, 6G). These results indicate that Sirt1 overexpression in MSCs can accelerate skeletal muscle injury repair by promoting regeneration.

### 1,25(OH)_2_D_3_ up-regulates the expression of Myod1 in C2C12 cells through VDR-mediated transcription

**Figure 7:**
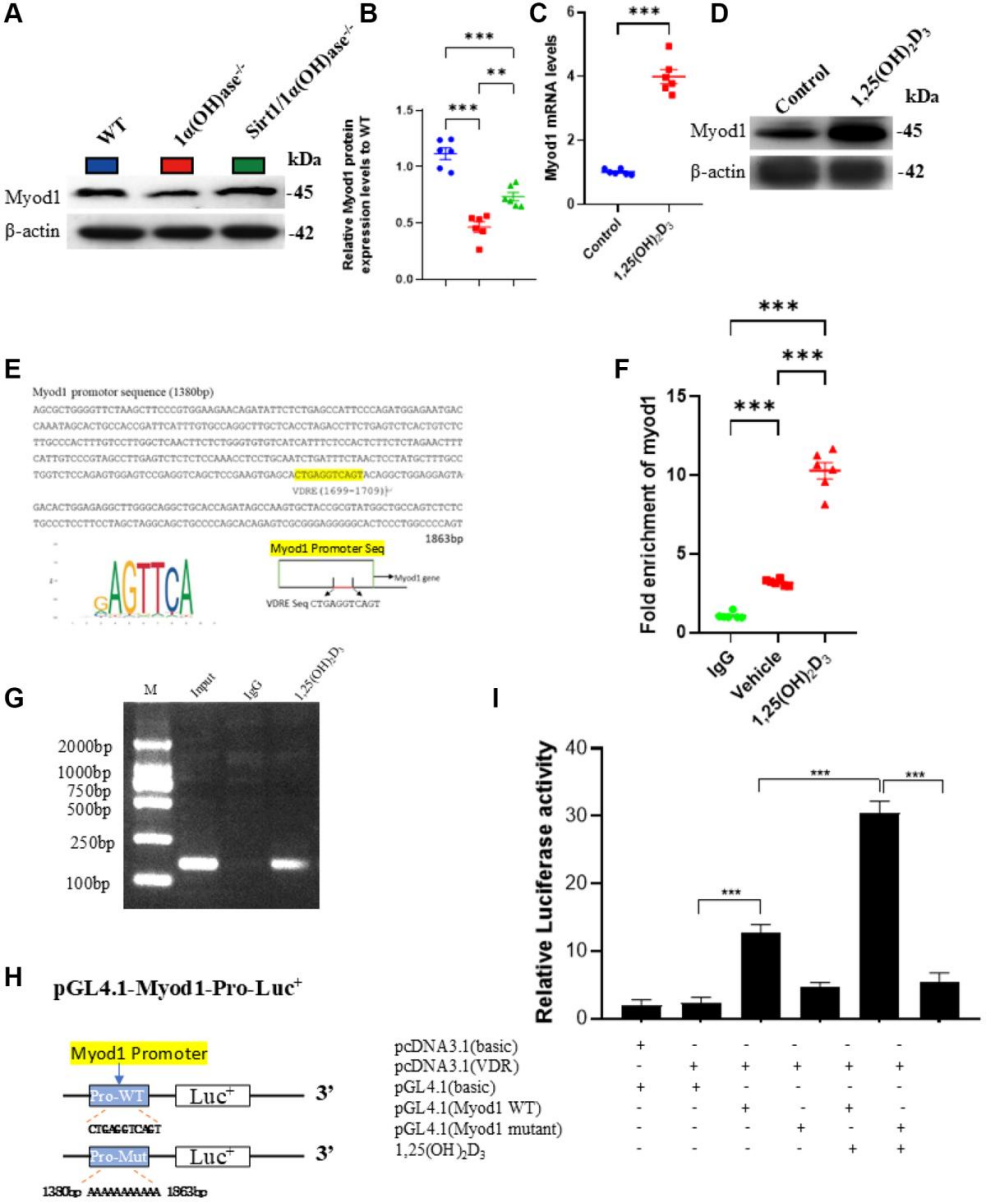
**1,25(OH)_2_D_3_ up-regulates the expression of Myod1 in C2C12 cells through VDR-mediated transcription.** (**A**) Western blot showing changes in Myod1 protein levels in tibialis anterior muscles of 2-month-old WT, 1α(OH)ase^−/−^ and Sirt1^Tg^1α(OH)ase^−/−^ mice and (**B**) quantitative analysis. (**C**, **D**) 10^−8^ M 1,25(OH)_2_D_3_ treatment upregulates Myod1 gene and protein expression in C2C12 cells. (Each experiment was conducted in five replicates). (**E**) Predicted VDR binding site (highlighted in yellow) in the Myod1 promoter region. (**F**) qPCR results showing enrichment of Myod1 in VDR chromatin immunoprecipitation. (**G**) Agarose gel electrophoresis of ChIP PCR products. (**H**) Schematic diagram showing construction of luciferase reporter plasmids containing the Myod1 promoter region. (**I**) Luciferase activity analysis. Each experiment was performed in triplicate. ^*^p < 0.05; ^***^p < 0.001, compared to control; ^#^*p* < 0.05; ^###^*p* < 0.001, compared to 1α(OH)ase^−/−^ mice or Vehicle.

Given that MyoD1 plays a very important role in promoting myoblast differentiation, myoblast fusion and myotube formation as well as in the regulation of muscle-specific gene expression during muscle regeneration in response to injury and atrophy, we utilized Western blotting to detect the levels of MyoD1 protein expression in tibialis anterior muscles of 2-month-old male wild-type, 1α(OH)ase^−/−^ and Sirt1^Tg^1α(OH)ase^−/−^ littermates. The results showed that MyoD1 protein expression was significantly downregulated in skeletal muscles of 1α(OH)ase^−/−^ mice, while it was markedly upregulated in skeletal muscles of 1α(OH)ase^−/−^ mice with MSC-overexpressing Sirt1 (Figure 7A, 7B). Previous studies showed that active vitamin D can upregulate MyoD1 expression in skeletal muscle, however the underlying mechanism has been unclear. We treated C2C12 cells with 10^−8^ M 1,25(OH)_2_D_3_ and detected Myod1 mRNA and protein levels using real-time RT-PCR and Western blot. The results showed that 1,25(OH)_2_D_3_ treatment significantly increased Myod1 mRNA and protein expression in C2C12 cells (Figure 7C, 7D). To investigate whether 1,25(OH)_2_D_3_ transcriptionally regulates Myod1 expression through VDR, we performed bioinformatic analysis and identified a VDRE-like sequence in the Myod1 gene promoter region (Figure 7E). We then utilized ChIP assay to examine whether VDR could bind to the VDRE in the Myod1 promoter. PCR detection of the ChIP products showed that the Myod1 promoter VDRE sequence was present in the total input sample, and its enrichment by VDR antibody immunoprecipitation was greater than that by IgG immunoprecipitation (Figure 7F, 7G). These results demonstrated that VDR could physically bind to the VDRE-like sequence in the Myod1 promoter region. To further confirm that 1,25(OH)_2_D_3_ transcriptionally regulates Myod1 expression through VDR, we constructed luciferase reporter plasmids containing the Myod1 promoter region and a VDR overexpression plasmid (Figure 7H). After transfection into C2C12 cells and 1,25(OH)_2_D_3_ treatment for 24 h, luciferase reporter assays were performed at 48 h. The results showed that compared to empty vector, luciferase activity significantly increased in C2C12 cells transfected with pGL4.1-Myod1 (WT) plasmid, and was further enhanced by 1,25(OH)_2_D_3_ treatment. However, luciferase activity did not increase in C2C12 cells transfected with a pGL4.1-Myod1 (mutant) plasmid, nor was the mutant reporter activated by 1,25(OH)_2_D_3_ treatment (Figure 7I). These results demonstrate that 1,25(OH)_2_D_3_ can upregulate Myod1 expression in C2C12 cells by VDR-mediated transcriptional regulation.

## DISCUSSION

Active vitamin D plays a critical role in maintaining optimal muscle function and health. Deficiency in this vital nutrient has been strongly linked to muscle atrophy, weakness, impaired regeneration, and notably, sarcopenia. These associations are supported by more recent studies which emphasize the health implications of vitamin D deficiency in muscle physiology [18, 19].

Our findings build on these contemporary insights by demonstrating, for the first time, that vitamin D modulates muscle mass and function through the regulation of Sirt1 expression and activity. Specifically, 1,25(OH)2D3 upregulated Sirt1 in muscle cells via the vitamin D receptor (VDR). This pathway’s disruption, as observed in our 1α-hydroxylase knockout mice, led to reduced Sirt1 expression, contributing to sarcopenia. The restoration of Sirt1 expression through overexpression strategies not only rescued the muscle phenotype but also highlighted the pivotal mediating role of Sirt1.

The preference for fast-twitch type II fiber atrophy is a recognized contributor to sarcopenia. Recent studies have suggested that strategies focusing on maintaining or restoring these fibers could be beneficial in mitigating age-related muscle wasting [20]. Our data extend this concept by showing that 1,25(OH)2D deficiency leads to muscle fiber atrophy and a reduction in glycolytic type II fibers, which was ameliorated by Sirt1 overexpression in mesenchymal stem cells (MSCs). This underlines the beneficial effects of 1,25(OH)2D on muscle and highlights the Sirt1 axis as a promising target for sarcopenia therapies.

Maintaining a robust muscle stem cell pool is essential for lifelong muscle function and health. Satellite cells are pivotal for this process. Our study aligns with recent findings which suggest that enhancing the regenerative capacity of satellite cells could provide a therapeutic avenue to combat sarcopenia [21]. We observed that 1,25(OH)2D deficiency depleted Pax7+ satellite cells, a loss that was countered by Sirt1 overexpression in MSCs. This suggests that Sirt1 not only supports the stem cell compartment but also enhances the muscle’s intrinsic regenerative capacity, offering a novel approach to counter muscle wasting.

Skeletal muscle senescence disrupts tissue structure and function [22]. Selectively removing senescent cells or inhibiting senescence associated secretory phenotype (SASP) offers therapeutic promise [23]. We previously reported that 1,25(OH)_2_D deficiency causes premature muscle aging [24]. Here 1,25(OH)_2_D deficient mice exhibited heightened senescence markers p16/p21 and SASP factors including p65 and IL-1α, indicative of cellular senescence. Overexpressing Sirt1 markedly decreased these senescent and pro-inflammatory markers. This suggests that Sirt1 activation suppresses muscle cell senescence and related inflammation, representing an attractive sarcopenia strategy.

Sirt1 reportedly inhibits cellular senescence by deacetylating and reducing p53 transcriptional activity [25]. It also deacetylates nuclear NF-kB/p65 to curb inflammation [26]. We found 1,25(OH)_2_D_3_ and the Sirt1 agonist resveratrol inhibited muscle cell senescence and SASP by reducing acetylated, nuclear p53 and p65 levels and function. Immunofluorescence revealed 1,25(OH)_2_D_3_ and resveratrol treatment retained p53/p65 in the cytoplasm. This indicates Sirt1 stimulation suppresses muscle aging and inflammation by deacetylating and inhibiting p53 and p65.

Sirt1 promotes muscle repair after injury by suppressing senescence and inflammation while activating precursor cells [27]. We showed Sirt1 overexpression in MSCs markedly enhanced regeneration following muscle damage in mice. It increased embryonic myosin expression and proliferation while lowering p53, p65 and inflammation. This demonstrates that stem cell-mediated Sirt1 augmentation accelerates injury repair by stimulating precursor cells and regeneration.

Myod1 is known to play a critical role in myoblast differentiation and fusion into myotubes during muscle regeneration [28]. Our results show first that Myod1 protein levels are reduced in muscles of 1α(OH)ase^−/−^ mice, and rescued in 1α(OH)ase^−/−^ mice overexpressing Sirt1 in MSCs. This suggests 1,25(OH)_2_D signaling regulates Myod1 expression *in vivo*. Further mechanistic studies in C2C12 cells revealed that 1,25(OH)_2_D_3_ treatment increases Myod1 mRNA and protein expression. Bioinformatic analysis identified a putative VDRE in the Myod1 promoter. Chromatin immunoprecipitation experiments demonstrated VDR directly binds this Myod1 promoter VDRE region. Additionally, luciferase reporter assays using a construct with the Myod1 promoter showed 1,25(OH)_2_D_3_ treatment enhances luciferase activity, while mutation of the VDRE prevents this induction. Taken together, these comprehensive data provide strong evidence that 1,25(OH)_2_D_3_ transcriptionally upregulates Myod1 by facilitating VDR binding to the Myod1 promoter. This highlights a novel mechanism by which active vitamin D signaling promotes myogenic differentiation and muscle regeneration, through direct modulation of the master regulator myogenic transcription factor, Myod1.

It is important to note the potential interaction between bone and muscle effects in our model system. Our previous work demonstrated that Sirt1 overexpression significantly improved osteopenia in 1α-hydroxylase-deficient mice [16]. The bone-muscle crosstalk, mediated through various endocrine and paracrine factors, may contribute to the observed improvements in muscle mass. The skeletal system serves not only as a mechanical support but also as an endocrine organ, producing factors such as osteocalcin and prostaglandin E2, which can influence muscle metabolism and function [29, 30]. Therefore, the protective effects of Sirt1 overexpression on muscle mass observed in our study may be partially mediated through improved bone health. This bone-muscle interaction is particularly relevant in the context of vitamin D deficiency, as 1,25(OH)_2_D_3_ plays crucial roles in both tissues [31]. Future studies employing tissue-specific deletion models could help delineate the direct effects of Sirt1 on muscle versus indirect effects mediated through improved bone metabolism [32]. Understanding these tissue-specific and systemic interactions will be crucial for developing targeted therapeutic strategies for both sarcopenia and osteoporosis in vitamin D-deficient conditions.

While our study demonstrates the protective effects of MSC-specific Sirt1 overexpression on muscle mass and fiber composition in vitamin D-deficient mice, several limitations should be acknowledged. Notably, we did not assess muscle strength parameters such as grip strength or functional measures like locomotor activity, which are crucial diagnostic criteria for sarcopenia in both clinical settings and experimental models. This is particularly relevant as previous studies have shown that vitamin D deficiency may have a more pronounced impact on muscle strength and function compared to muscle mass alone [24, 33]. Indeed, clinical evidence suggests that improvements in vitamin D status often correlate more strongly with functional outcomes than with changes in muscle mass [34]. Future studies should incorporate these functional assessments to provide a more comprehensive understanding of how Sirt1 overexpression affects not only muscle mass but also muscle strength and performance in the context of vitamin D deficiency. Additionally, investigation of specific force production at the single fiber level would provide valuable insights into the quality of muscle function beyond mass-related parameters [35].

In summary, the results of this study indicate that deficiency of active vitamin D down-regulates Sirt1 and Myod1 through reduced VDR-mediated gene transcription, increases p53 and p65 acetylation levels, inhibits the proliferation and differentiation of skeletal muscle precursor cells and skeletal muscle cell regeneration, and increases skeletal muscle cell senescence and SASP, thereby accelerating the occurrence of sarcopenia, while the overexpression of Sirt1 in MSCs can prevent the occurrence of sarcopenia caused by active vitamin D deficiency. This study elucidates novel vitamin D molecular mechanisms in muscle and highlights Sirt1 as a potential key therapeutic target for age-related muscle wasting. It provides an experimental basis for exploiting active vitamin D and Sirt1 activation clinically to prevent sarcopenia.

## MATERIALS AND METHODS

### Animals

Three mouse models were used: 1) Sirt1^Tg^ mice expressing elevated Sirt1 under the 2.4 kb Prx1 promoter [36]; 2) 1α(OH)ase^−/−^ mice generated by breeding 1α(OH)ase^+/−^ heterozygotes [37], and 3) Sirt1^Tg^1α(OH)ase^−/−^ mice generated by crossing double mutants. All mice were on a C57BL/6J background. 1α(OH)ase^−/−^ mice were fed a rescue diet [38]. Two-month-old male wild-type (WT), Sirt1^Tg^, 1α(OH)ase^−/−^ and Sirt1^Tg^1α(OH)ase^−/−^ littermates were used. All procedures were approved by the Institutional Animal Care and Use Committee of Nanjing Medical University (protocol No. IACUC2002016). All animal experiments mentioned in this manuscript were conducted in The Research Center for Bone and Stem Cells at Nanjing Medical University, under the supervision of Professor Dengshun Miao. The experiments were performed by Biqi Ren, Jing Wang, and Xingchen Liu, all of whom are members of Professor Miao’s laboratory.

### Histology and immunofluorescence staining

After euthanasia, the tibialis anterior (TA) muscle was removed from the lower limbs, frozen and embedded under liquid nitrogen using OCT, and then cut into 5 µm sections on a cryostat. After rewarming the muscle sections at room temperature, they were stained with hematoxylin and eosin (H&E). For immunofluorescence (IF) staining, the sections were fixed in acetone, incubated with 3% H_2_O_2_, and then the slides were incubated with antibodies against laminin (Proteintech, China, 1:200), MyHC IIA (cat. #SC-71; DSHB, USA, 1:100), MyHC IIB (cat. #BF-F3; DSHB, 1:100), PAX7 (cat. #Pax7; DSHB, 1:100), p16 (Abcam, USA, 1:200) and p21 (Abcam, USA, 1:200), p65 (Abmart, China, 1:200), Aceyl-p53 (Abcam, UK, 1:200) and Aceyl-p65 (Abcam, UK, 1:200), eMyHC (cat. #F1.652; DSHB, 1:100) and BrdU (Santa Cruz, USA, 1:100) were incubated overnight at 4°C. They were then incubated with the secondary antibodies for staining. Slides were mounted with mounting medium containing DAPI (Sigma-Aldrich, St. Louis, MO, USA), and images were taken with a fluorescence microscope (Leica, Wetzlar, Germany).

### RNA isolation and real-time RT-PCR

Total RNA was extracted from C2C12 cells using Trizol reagent (Invitrogen, Carlsbad, CA, USA) according to the manufacturer’s instructions. Complementary DNA (cDNA) was synthesized using Synthesis SuperMix (Invitrogen). Real-time RT-PCR was carried out using an Agilent Real-time System as described previously [39]. Gapdh was amplified at the same time to normalize gene expression. Each experiment was repeated three times to determine relative gene expression differences. The sequence-specific primers of human and mice are displayed in Table 1.

**Table 1:** Primers used for quantitative real-time PCR.

**Primers**	**Forward**	**Reverse**
**Sirt1**	GCTGACGACTTCGACGACG	TCGGTCAACAGGAGGTTGTCT
**Myodl**	CCACTCCGGGACATAGACTTG	AAAAGCGCAGGTCTGGTGAG
**Gapdh**	CCACCCAGAAGACTGTGGAT	GGATGCAGGGATGATGTTCT

### Western blotting

Tissue or cell lysates were extracted for loading into 10% SDS-PAGE gels and immunoblotting was performed as previously described [16]. Primary antibodies, including Sirt1 (Millipore, 07-131, 1:1000), p16 (Proteintech, 10883-1-AP, 1:1000), p21 (sc-471, Santa Cruz Biotechnology, 1:500), p53 (#2524, Cell Signaling Technology, 1:1000), p65 (Abmart, China, 1:1000), IL-1α (ab7632, Abcam, UK, 1:500), IL-6 (ab6672, Abcam, UK, 1:1000), MMP3 (ab52915, Abcam, UK, 1:1000), β-Actin (Cell Signaling Technology, 8457S, 1:2000) were used for immunoblotting. Immunoreactive bands were visualized with ECL chemiluminescence (Bio-Rad, Hercules, CA, USA) and analyzed by ImageJ.

### Immunoprecipitation

Immunoprecipitation experiments were carried out by using Pierce^™^ Crosslink Magnetic IP Kit (Thermo Fisher Scientific, Waltham, MA, USA). An immunoprecipitation assay was performed as recommended by the supplier. Proteins extracted from 2 × 10^6^ mouse myogenic C2C12 cells were mixed with 1 µg of Sirt1 antibody and prewashed Protein A/G, then incubated overnight. The bound antigens were eluted from the beads by boiling samples for 10 min. Eluted samples were obtained from SDS-PAGE. Immunoblotting was carried out as previously described [16]. Proteins were extracted from C2C12 cells. Primary antibodies against Sirt1, Aceyl-p53 and Aceyl-p65 (Cell Signaling Technology, Danvers, MA, USA) were used. The immunoreactive bands were visualized by ECL chemiluminescence (Amersham, UK).

### ChIP-qPCR

C2C12 cells were cultured to perform chromatin immunoprecipitation (ChIP) analyses of VDR recruitment by using an anti-VDR antibody (Abcam) and SimpleChIP^®^ Enzymatic Chromatin IP Kit (Cell Signaling Technology, Danvers, MA, USA). ChIP analyses were performed as recommended by the supplier to identify the VDR recruitment. Immunoprecipitation was performed using either a control IgG or rabbit anti-VDR antibody (Abcam, ab3508). The co-precipitated chromatin was determined by qPCR for the presence of human Sirt1 promoter sequence using Sirt1 sense 5′-GGCTTAGAGTTGTGAAGGAACTA-3′ and antisense 5′-GACATGTATGGAGCCAAAAGACC-3′ primers or for the presence of human Myod1 promoter sequence using Myod1 sense 5′-GTCAGCTCCGAAGTGAGCA-3′ and antisense 5′-CGCCTCAAGCCAATAGGAGT-3′ primers. The PCR products were electrophoresed on 2% agarose gels, and visualized by ethidium bromide staining.

### Construction of promoter-reporter plasmids and dual-luciferase transient expression assay

Analysis of the transcriptional activity of the VDR was conducted. The full coding sequence of VDR was amplified and cloned into a pCDNA3.1 vector used as an effector vector. The promoters of the Sirt1 or Myod1 gene were cloned into the GV238-LUC reporter vector. The plasmid pGL4.1-Sirt1 containing -ctgattcaag- in the promoter region of the mouse Sirt1 gene linked to the promoter-less firefly luciferase gene, and a mutational plasmid pGL4.1-Sirt1-mut in which -ctgattcaag- was changed into - aaaaaaaaaa-, were constructed. The plasmid pGL4.1-Myod1 containing -ctgaggtcagt- in the promoter region of the mouse Sirt1 gene linked to the promoter-less firefly luciferase gene, and a mutational plasmid pGL3-Sirt1-mut in which -ctgaggtcagt- was changed into -aaaaaaaaaa-, were constructed. C2C12 cells were plated in 24-well Falcon plates at a density of 100,000 cells/well in α-MEM with 10% FBS 24 hours prior to transient transfection. Plasmids were transfected in individual wells using Opti-MEM and liposome according to the manufacturer’s protocol. Briefly, each well was treated with pcDNA3.0-VDR and pGL4.1-Sirt1 or pGL4.1-Sirt1-mut or with pcDNA3.0-VDR and pGL4.1-Myod1 or pGL4.1- Myod1-mut (with or without 10^−8^M 1,25(OH)_2_D_3_), along with 40 ng of Renilla reniformis luciferase. The Renilla reniformis luciferase plasmid allows for constitutive, low-level expression to monitor DNA transfection efficiency. Forty-eight hours post-transfection, the cells were lysed in 1× passive lysis buffer (Promega, Madison, WI, USA) and the lysates were collected. Each lysate was then analyzed sequentially for Firefly and Renilla luciferase activity using a Dual-Luciferase Assay Kit (Promega Corp., Madison, WI, USA). All operating procedures followed the instructions provided by the reagent kit. The mean ratio of Firefly/Renilla for 6 biological replicates (wells) was calculated for each experimental treatment group.

### C2C12 cell cultures

The mouse myogenic C2C12 cell line was obtained from ATCC and cells were used up until passage number 8. Myoblasts were maintained on plastic cell culture dishes in Dulbecco’s modified Eagle’s medium (DMEM) supplemented with 10% fetal bovine serum and 1% penicillin-streptomycin in a humidified incubator kept at 37°C and 5% CO_2_. When cells reached 70% confluency, they were cultured in the absence or presence of 100 µM H_2_O_2_ or 10 µM resveratrol or 10^−8^M 1,25(OH)_2_D_3_ for 24 hrs. Cells were then stained by immunofluorescence for Aceyl-p53 and Aceyl-p65.

### Statistical analysis

Results are expressed as mean ± s.e.m. Statistical analysis was performed using GraphPad Prism 5 software (GraphPad Software Inc., San Diego, CA, USA). Comparisons between two groups were analyzed using a two-tailed unpaired Student’s *t*-test. Comparisons among three or more groups were performed using two-way ANOVA followed by Dunnett’s postdoc multiple comparisons. *P*-values < 0.05 were considered statistically significant.
